# Comprehensive characterisation of pulmonary and serum surfactant protein D in COPD

**DOI:** 10.1186/1465-9921-12-29

**Published:** 2011-03-11

**Authors:** Carla Winkler, Elena N Atochina-Vasserman, Olaf Holz, Michael F Beers, Veit J Erpenbeck, Norbert Krug, Stefan Roepcke, Gereon Lauer, Martin Elmlinger, Jens M Hohlfeld

**Affiliations:** 1 Department of Clinical Airway Research, Fraunhofer Institute for Toxicology and Experimental Medicine, Hannover, Germany; 2 Department of Respiratory Medicine, Hannover Medical School, Hannover, Germany; 3 Division of Pulmonary, Allergy, and Critical Care Medicine, Department of Medicine, University of Pennsylvania School of Medicine, Philadelphia, PA, USA; 4 Nycomed GmbH, Konstanz, Germany

## Abstract

**Background:**

Pulmonary surfactant protein D (SP-D) is considered as a candidate biomarker for the functional integrity of the lung and for disease progression, which can be detected in serum. The origin of SP-D in serum and how serum concentrations are related to pulmonary concentrations under inflammatory conditions is still unclear.

**Methods:**

In a cross-sectional study comprising non-smokers (n = 10), young - (n = 10), elderly smokers (n = 20), and smokers with COPD (n = 20) we simultaneously analysed pulmonary and serum SP-D levels with regard to pulmonary function, exercise, repeatability and its quaternary structure by native gel electrophoresis. Statistical comparisons were conducted by ANOVA and post-hoc testing for multiple comparisons; repeatability was assessed by Bland-Altman analysis.

**Results:**

In COPD, median (IQR) pulmonary SP-D levels were lower (129(68) ng/ml) compared to smokers (young: 299(190), elderly: 296(158) ng/ml; p < 0.01) and non-smokers (967(708) ng/ml; p < 0.001). The opposite was observed in serum, with higher concentrations in COPD (140(89) ng/ml) as compared to non-smokers (76(47) ng/ml; p < 0.01). SP-D levels were reproducible and correlated with the degree of airway obstruction in all smokers. In addition, smoking lead to disruption of the quaternary structure.

**Conclusions:**

Pulmonary and serum SP-D levels are stable markers influenced by smoking and related to airflow obstruction and disease state. Smaller subunits of pulmonary SP-D and the rapid increase of serum SP-D levels in COPD due to exercise support the translocation hypothesis and its use as a COPD biomarker.

**Trial registration:**

no interventional trial

## Introduction

Chronic obstructive pulmonary diseases (COPD) is a multi-component disease. It is characterized by airflow limitation that is not fully reversible when treated with bronchodilators. In COPD an abnormal airway inflammatory response, a thickening of airway walls, destruction of alveoli and the enlargement of air spaces can be observed [1]. Tobacco smoking is the primary cause and major risk factor for the development of COPD and in most industrialized countries the disease has an increasing prevalence [2].

SP-D is synthesized in type II pneumocytes and Clara cells. It is composed of monomers (43 kDa), which assemble into trimers via disulfid crosslinking and undergo further multimerization to higher order such as dodecamers and oligomers (~ 1 MDa) [3]. Each monomer has four distinct domains: the carbohydrate recognition domain (CRD), the neck domain, a collagenous domain and the N-terminal cystein-rich domain. The integrity of the quaternary structure is important for functions such as in pulmonary surfactant and lipid homeostasis [4], innate immunity [3], regulation of cellular clearance as well as inflammatory and immune responses [5]. Importantly, destruction of the quaternary structure leads to reduced binding affinity of the CRD to pathogens or allergens [6,7] and can promote a switch towards pro-inflammatory signalling [8,9].

SP-D can be detected in serum and increased serum levels have been reported for lung diseases such as pulmonary alveolar proteinosis, cystic fibrosis, COPD, and for infectious diseases like tuberculosis and bacterial pneumonia [10-12]. Lomas et al. also report an association between high serum SP-D levels and an increased risk for COPD exacerbations [12]. These data suggest that SP-D levels in serum reflect disease activity and SP-D has therefore been suggested as a potential biomarker for the epithelial integrity in COPD.

The precise mechanism leading to increased serum levels is unclear. Based on the currently most widely accepted hypothesis, SP-D translocates from the lung into the blood, a process that could be regulated by changes in the alveolar-capillary permeability [13]. However, the relationship between concentrations in serum and bronchoalveolar lavage fluid (BAL) is different for allergic diseases like asthma and for smokers or patients with COPD. In asthma or allergen induced airway inflammation increased levels of SP-D were detected in both BAL [14] and serum [15], compatible with the notion that a higher concentration in one compartment also leads to a higher concentration in the other. For smokers and especially for COPD patients reduced levels of SP-D were detected in BAL, however, both groups also show elevated concentrations of SP-D in serum [12]. In line with this, higher levels of SP-D were observed in BAL of patients under steroid treatment [16], while treatment with oral steroids leads to a decline in serum to SP-D concentrations of COPD patients [12].

However, despite these advances, the utility of SP-D as a biomarker has not yet been fully realized due to several factors: 1) A complete characterization of SP-D expression in both compartments (BAL and serum) from healthy controls, smokers or COPD patients has been lacking; 2) Oxidative-nitrative stress and the action of proteases are both increased in smokers and COPD patients [1] and have been shown to modify the quaternary structure of SP-D [17,18] thus potentially affecting accurate measurement; 3) Although SP-D was shown to be unaffected by physical exercise in healthy volunteers [19], the effect on exercise on these parameters in disease states is largely unknown.

Based on this we embarked on a comprehensive characterization of SP-D expression in controls, smokers and patients with COPD. We hypothesized that due to changes in barrier integrity and molecular sizing, lower SP-D levels in BAL would be associated with higher concentrations of SP-D in serum in smokers and especially in COPD patients. In addition to assessment of the overall concentration of SP-D in two compartments, we also assessed the quaternary structure of SP-D in BAL samples and measured SP-D levels in serum samples obtained before, during and after a moderate exercise period.

## Materials and methods

### Study subjects

Peripheral blood and BAL was investigated from subjects of two different studies. Both studies were performed in accordance with Good Clinical Practice and the Declaration of Helsinki. Subjects gave their written consent after being fully informed about the purpose and nature of the study. The studies were approved by the Ethical Committee of Hannover Medical School.

#### Study in healthy non-smokers (H) and young smokers (S1)

Ten non-smokers (21 - 36 years, 3 male) and ten smokers (21 - 49 years, 7 male) with no history of allergic or other diseases were enrolled into the study. Only subjects with forced expiratory volume in 1 sec (FEV_1_) > 77% of predicted normal and a ratio of FEV_1_/forced vital capacity (FVC) > 70%, normal findings in electrocardiogram, differential blood cell count, blood coagulation, and serum parameters (gamma-glutamyl-transferase, aspartate aminotrans-ferase, alanine aminotransferase, urea, creatinine, sodium, potassium, IgE) as well as negative skin-prick test for 15 standard allergens (ALK-SCHERAX Arzneimittel GmbH, Hamburg, Germany) were included. None of the subjects suffered from an acute bronchitis 4 weeks prior to bronchoscopy. Non-smokers were required not to have smoked for at least five years. An inclusion criterion for smokers was a minimum consumption of 15 cigarettes per day for at least two years. Levels of cotinine were measured to prove presence or absence of nicotine exposure.

After a screening visit, blood sampling and bronchoscopy with BAL was performed during a single visit. Subjects were discharged from the study following a termination visit 1 - 7 days after the procedures.

#### Study in elderly smokers (S2) and smokers with COPD (C)

Forty current smokers (40 - 75 years) with at least 10 pack years and active smoking confirmed by urine cotinine measurement were enrolled. One half of the group (n = 20) had normal pulmonary function (FEV_1_/FVC ≥ 70% and FEV_1 _≥ 85% pred.) while the other subjects (n = 20) had COPD GOLD stage 2 (Global Initiative for Chronic Obstructive Lung Disease) with typical clinical characteristics (cough and sputum) and with a post-bronchodilator FEV_1_/FVC < 67% and 50% ≤ FEV_1_< 75%. The groups were matched for age and gender (6 female/14 male) and no subject suffered from a respiratory tract infection or recent exacerbation of COPD (within 4 weeks prior to screening examination). None of the subjects had a history or evidence of clinically relevant allergies, evidence of any disease that would have affected the subject's safety during study participation, particularly during bronchoscopy and exercise testing including pulmonary, hepatic, renal (creatinine above 2 mg/dL), gastrointestinal, haematological, endocrinological, metabolic, neurological, psychiatric, or cardiovascular disorders, particularly arterial hyper- or hypotension, symptomatic coronary heart disease (i.e. angina pectoris induced by stress or physical effort), congestive heart failure (New York Heart Association functional classification III and IV), and cardiac arrhythmia. Furthermore, subjects with chronic inflammatory disease other than COPD, diagnosis of cancer within 5 years of study start, evidence of drug or alcohol abuse, or regular intake of theophylline, lipoxygenase inhibitors, leukotriene antagonists, inhaled and oral cromones, systemic glucocorticosteroids, inhaled and topical glucocorticosteroids, anti-TNF-α agents, hormonal contraceptives, and nitrates were not eligible for participation.

The study was composed of two pairs of consecutive visits: visit 1 and 2 followed 28 ± 5 days later by visit 3 and 4. Visit 2 and visit 4 was performed 3 to 7 days after visit 1 and visit 3, respectively. Bronchoscopy and blood sampling was performed on visit 2 and 4, serum sampling during constant load exercise was performed on visit 1 and 3. Lung function measurements were performed at screening prior to visit 1. A termination visit with discharge of the subject from the study was performed 1 - 4 days after visit 4.

### Exercise

Subjects with COPD and elderly smokers first performed a screening exercise test to determine the peak work capacity. After a one minute warm-up consisting of load less pedalling, a stepwise increase in the work rate of 10 watts every minute, starting at 10 watts followed. Pedalling rates were kept within 50-70 rpm throughout exercise. Exercise continued until the subject felt tired, was unable to maintain a pedalling frequency of at least 40 rpm, or if exercise could not be continued safely. Peak work capacity (W_peak_) was defined as the highest work rate that could be maintained for at least 30 seconds. The constant load exercise test was conducted at the indicated time points. After warm up, the work rate was increased to 75% of W_peak_. The subject was encouraged to exercise for as long as possible, but the maximum time was limited to 30 minutes.

### Bronchoscopic procedure and processing BAL cells

The bronchoscopic procedure and the processing of BAL fluid and cells was performed as described before [20]. Briefly, fiberoptic bronchoscopy was done with standard premedication under topical anesthesia to allow collection of BAL (5 × 20 mL of sterile saline plus initial 20 mL discard). BAL cells were filtered through a 100-μm filter, centrifuged at 250 *g *for 10 min, and resuspended in phosphate-buffered saline (PBS). The total count of nucleated cells was performed using a Neubauer hemocytometer. Differential cell counts were performed from cytospin slides, with 300 cells per slide being counted.

Protein content in BAL and serum was determined according to the method of Bradford [21].

### Serum sampling

Blood was collected in a S-Monovette^®; ^(Sarstedt, Nuembrecht, Germany), allowed to stand for 30 min, and then centrifuged for 15 min with 1600 × g. Serum was aliquoted and kept frozen at -80°C until analysis.

### Reagents

All reagents for electrophoresis and immunoblotting were purchased from Invitrogen, Carlsbad, CA, USA unless otherwise specified.

Anti SP-D antibody (Ab #3434) was purchased from Chemicon, Temecula, CA, USA and a polyclonal antibody against SP-D (Ab 1754) was produced as previously described [22].

### Measurement of surfactant protein D in BAL and serum

SP-D levels in serum and BAL samples were measured with a colorimetric sandwich assay in duplicates (BioVendor, Heidelberg, Germany) according to the manufacturer's instruction.

### Polyacrylamide Gel Electrophoresis and Immunoblotting for SP-D

BAL proteins were separated and analyzed by denaturating SDS PAGE and immunoblotting as described before [22].

Native gel electrophoresis for detection of the quaternary SP-D structure was performed according to Schagger [23] using NativePAGE 4-16% Bis-Tris gels. Briefly, equal amounts of SP-D (as determined using SDS PAGE) were mixed with cold native sample buffer before loading. Electrophoresis was run at room temperature at a constant voltage of 150 V for 2 h. Immunoblotting and detection of SP-D was performed as described above.

### Oxidative modification of SP-D *in vitro*

Recombinant rat SP-D was produced in CHO cells as described previously [24]. For *in vitro *oxidative modification of rrSP-D (10 μg/ml) and BAL proteins, incubation with the synthetic oxidizing agent AAPH (2,2-Azo-bis-(2-amidinopropane)-dihydrochloride, Sigma, Taufkirchen, Germany) 74 mM for 2 h at 37°C was performed as described before [18]. Modifications were analyzed by blue native gel electrophoresis.

### Statistical analysis

**Figure 1:**
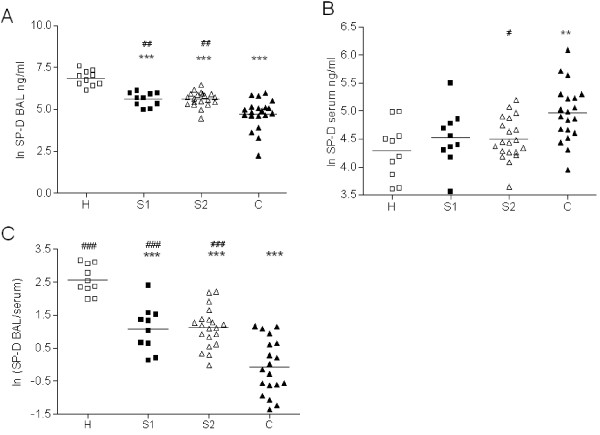
**SP-D levels in BAL and Serum**. SP-D levels in BAL (A) serum (B) and the ratio of BAL/serum SP-D levels (C) of healthy, non-smoking subjects (H, open squares n = 10), young smokers (S1, black squares, n = 10), elderly smokers (S2, open triangles, n = 20) and smokers with COPD (C, black triangles n = 20). Log-transformed individual data points are provided together with the respective mean of the log transformed data. Values are displayed on a logarithmic scale. *p < 0.05, **p < 0.01, ***p < 0.001 compared to H, ^#^p < 0.05, ^##^p < 0.01 compared to C.

**Figure 2:**
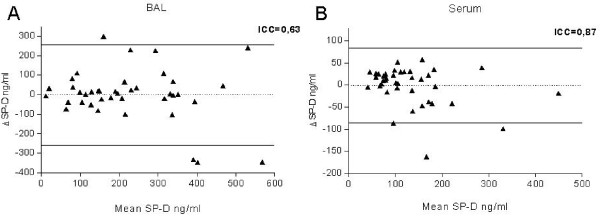
**Repeatability of SP-D measurements**. Bland-Altman plot to assess the repeatability of SP-D levels in BAL (A) and serum (B) of elderly smokers (S2) and smokers with COPD between two samplings with a time delay of about 34 ± 10 days. The coefficient of reliability (derived from one-way ANOVA as the ratio of variance among subjects to total variance) is given as intraclass correlation coefficient (ICC).

**Figure 3:**
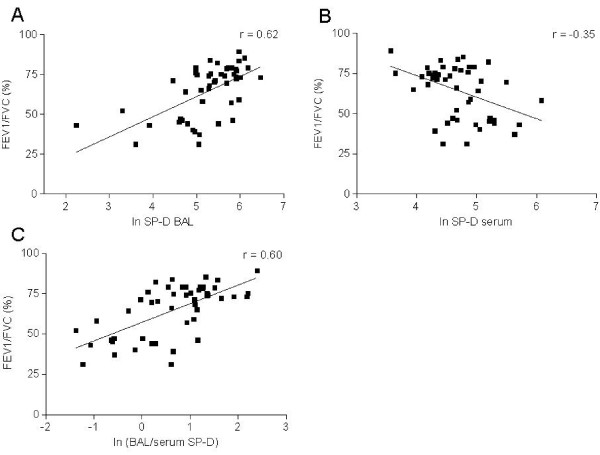
**Correlation of lung function measurements and SP-D levels**. Correlation of SP-D levels in BAL (A), serum (B) or the ratio of SP-D in BAL and serum (C) with FEV_1_/FVC in smokers (n = 50, young and elderly smokers and smokers with COPD). FEV_1_: forced expiratory volume in 1 s, FVC: forced vital capacity. Correlations were significant with p < 0.001, r = 0.62 (A); p = 0.013, r = 0.-0.35 (B) and p < 0.001, r = 0.60 (C).

Statistical analysis was performed by SAS (Cary, NC, USA) and Statistica (Statsoft, Hamburg, Germany). Comparisons between subject groups of clinical data and SP-D levels were conducted by ANOVA and post hoc testing for multiple comparison with Newman-Keuls (Table 1 and 2) and Tukey-Kramer (Figure 1), respectively. Analysis was performed after log-transformation for non-normal distributed data. Repeatability was assessed by Bland-Altman analysis. Intraclass correlation coefficients were derived from one-way ANOVA as the ratio of variance among subjects to total variance (Figure 2). Correlations were conducted by linear regression analysis according to Spearman (Figure 3). Values are given as mean ± SEM or median and quartiles, depending on data distribution. A p-value < 0.05 was considered as statistically significant.

**Table 1:** Clinical characteristics

subjects	age	gender	FEV_1_ [% predicted]	FEV_1_/FVC [%]	Pack years
healthy non-smokers (n = 10)	25.8 ± 1.4^#^	7 F, 3 M	106.1 ± 3.0^#^	79.8 ± 2.1^#^	0^#^
young smokers (n = 10)	30.7 ± 2.9^#^	3 M, 7 F	97.8 ± 3.9^#^	78.8 ± 2.0^#^	14.9 ± 4.4^#^.
elderly smokers (n = 20)	52.8 ± 1.4*	14 M, 6 F	112.8 ± 3.3^#^	74.7 ± 1.0^#^	38.6 ± 5.5*
smokers with COPD (n = 20)	55.0 ± 1.4*	14 M, 6 F	60.5 ± 1.5*	46.9 ± 2.2*	48.0 ± 2.7*

(F = female, M = male, FEV1 = forced expiratory volume in one second, FVC = forced vital capacity. Mean values ± SEM are given. * indicates p < 0.05 compared to healthy non-smokers and # indicates p < 0.05 to smokers with COPD.)

**Table 2:** Bronchoalveolar lavage data

Group	Recovery	Total Cells	Macrophages		Neutrophils		Eosinophils		Lymphocytes	
	
	*(ml)*	*(×10^3^/ml)*	*(×10^3^/ml)*	*(%)*	*(×10^3^/ml)*	*(%)*	*(×10^3^/ml)*	*(%)*	*(×10^3^/ml)*	*(%)*
*H*	75.0 ± 3.1	65.7 ± 5.5	58.7 ± 5.1	89.5 ± 0.9	1.4 ± 0.2	2.2 ± 0.3	0.4 ± 0.2	0.6 ± 0.3	4.8 ± 0.7	7.4 ± 1.1
*S1*	73.8 ± 3.2	220.1 ± 54.7*	197.3 ± 48.7*	89.8 ± 0.7	8.6 ± 2.4^#^	4.1 ± 0.7^#^	3.8 ± 2.6	1.1 ± 0.5	9.0 ± 1.8^#^	4.2 ± 0.2^#^

*S2*	68.3 ± 3.1	240.5 ± 33.7*	220.1 ± 31.7*	94.8 ± 0.7	3.1 ± 1.0	1.3 ± 0.4	1.2 ± 0.4	0.5 ± 0.1	3.3 ± 1.0	1.2 ± 0.2^#^
*C*	43.7 ± 3.7^#^	232.5 ± 37.4*	224.5 ± 29.2*	90.3 ± 4.5	2.0 ± 0.4	1.3 ± 0.5	2.0 ± 0.9	0.8 ± 0.3	1.9 ± 0.7*	0.7 ± 0.2^#^

Fluid recovery and cell counts (absolute and percentage of cells) in bronchoalveolar lavage of healthy, non smoking subjects (H) young smokers (S1), elderly smokers (S2) and smokers with COPD (C). Values are given as means ± SEM; * indicate p < 0.05 and ^# ^p < 0.01 compared to healthy non-smokers (H).

## Results

### Basic data and clinical characteristics

Basic data and clinical characteristics of the study subjects are given in Table 1. In smokers with COPD, lung function was lowest and pack years were highest compared to all other groups. The two groups of smokers (S1 and S2) had similar lung function, but elderly smokers had more pack years compared to young smokers. Patients with COPD and elderly smokers were matched for gender, while females dominated in the healthy non-smoking group and males were more prevalent in the group of young smokers.

### BAL recovery and differential cell count

As shown in Table 2, BAL fluid recovery was significantly reduced in patients with COPD. Cell numbers per mL BAL were found elevated in all smokers compared to healthy non-smokers, which was due to an elevation of the macrophage population. In young smokers neutrophils were found to be significantly increased compared to non-smokers. In patients with COPD, the number of lymphocytes was significantly reduced. No significant differences in total and differential cell count were seen between young smokers and elderly smokers.

### SP-D levels in BAL and serum

Total protein levels in BAL were not significantly different between groups (H: 74.1 ± 12.3 μg/ml, S1: 84.3 ± 12.2 μg/ml, S2: 78.1 ± 8.6 μg/ml, and C: 65.6 ± 5.6 μg/ml).

In contrast, SP-D levels in BAL were decreased in smokers and patients with COPD (Figure 1A).

While healthy, non-smokers had a median (quartiles) SP-D level in BAL of 967 (691;1399) ng/ml, SP-D was significantly reduced in BAL of young and elderly smokers (S1: 299 (205;395), p < 0.001; S2: 296 (213;371) ng/ml, p < 0.001) with no difference between these two. Importantly, SP-D levels in BAL of patients with COPD were lowest (129 (101;169) ng/ml, p < 0.001) giving a 7.5 fold reduction compared to healthy, non smoking subjects (H).

SP-D levels in serum (Figure 1B) showed an inverse relation between groups compared to BAL. While healthy subjects had the lowest SP-D levels in serum (76 (48;95) ng/ml) they were significantly elevated in smokers with COPD (140 (104;193) ng/ml, p < 0.01). No differences in SP-D serum levels between young smokers (S1) and elderly smokers (S2) were observed (88 (74;119) vs. 85 (71;123) ng/ml). In Figure 1C the ratio of SP-D in BAL and serum is shown. This ratio was significantly decreased in patients with COPD compared to healthy controls (median (quartiles): 0.8 (0.5;1.9) versus 11.6 (10.0;21.5), p < 0.001). The ratio in COPD patients was lower compared to smokers without lung function impairment.

### Reproducibility of SP-D levels in serum and BAL

To assess the potential of SP-D as a biomarker for COPD we tested the reproducibility of SP-D levels in BAL and serum within a group of 40 individuals comprising elderly smokers (S2, n = 20) and smokers with COPD (C, n = 20) (Figure 2). The mean time period between measurements was 32 ± 10 days.

The reproducibility was better for SP-D levels in serum (Figure 2B) compared to BAL (Figure 2A). The correlation coefficient was r = 0.76 for serum and r = 0.65 for BAL (both p < 0.001).

### SP-D ratio correlates to lung function in smoking subjects

A correlation of serum and BAL SP-D levels with the FEV1 (% pred.) of smoking subjects (S1, S2, C) was found (r = -0.34, p = 0.015, respectively r = 0.45, p = 0.006). In addition, there was a correlation of serum (r = -0.35, p = 0.013) and BAL SP-D levels (r = 0.62, p < 0.0001) with the degree of airway obstruction (FEV_1_/FVC) within the group of smokers (S1, S2, C, n = 50, Figure 3A, B) and in line with this a significant positive correlation was observed for the BAL/serum ratio (r = 0.60, p < 0.001). Consequently, the BAL/serum SP-D ratio declined with the worsening in lung function (Figure 3C).

### Exercise-induced alterations of serum SP-D levels

**Figure 4:**
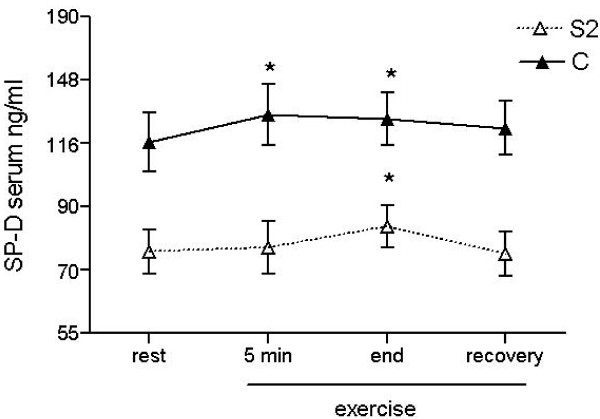
**Kinetics of SP-D levels in serum during constant load exercise**. Kinetics of SP-D levels in serum during constant load exercise taken at 4 different time points: at rest (rest), after 5 min of steady state exercise (5 min), at the end of loaded exercise (end), and 20 min after exercise (recovery). Change in SP-D serum concentrations over time in elderly smokers (S2, open triangles, dashed line, n = 15) and smokers with COPD (C, black triangles, n = 15) are shown. Data are given on the vertical axis on a logarithmic scale as mean ± SEM, * p < 0.05 compared to time point "rest".

We measured SP-D levels in serum during constant load exercise in elderly smokers (S2, n = 15) and smokers with COPD (C, n = 15) (Figure 4). In line with the findings under resting conditions, SP-D levels in serum of elderly smokers were significantly lower during exercise compared to patients with COPD (p < 0.001). Interestingly, we observed a significant increase change in serum SP-D levels as early as 5 min after the start of the exercise in patients with COPD (p < 0.05). A comparable change was also observed in the group of smokers, although to a later time point. When the exercise test was repeated, a comparable kinetic of changes was detected.

### Smoking alters the quaternary structure of SP-D

**Figure 5:**
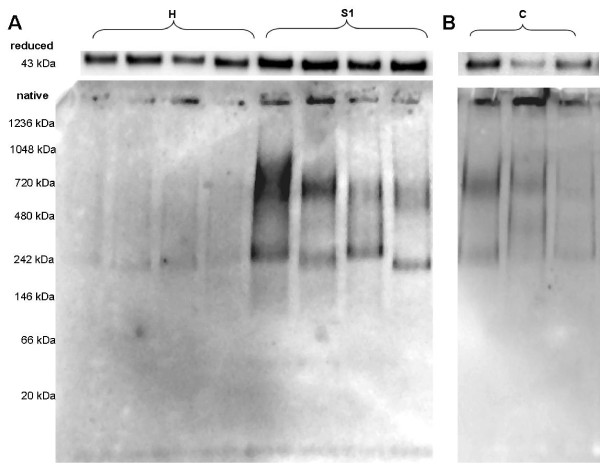
**Quaternary structure of pulmonary SP-D**. The quaternary structure of SP-D in BAL: A) equal amounts of SP-D were loaded as indicated by equal band intensities of the SP-D monomer (43 kDa) in SDS page under reduced conditions and immunoblotting (upper panel A and B). The quaternary structure of SP-D in bronchoalveolar lavage from 4 healthy subjects (H) and smokers (S1) is demonstrated by blue native electrophoresis and immunoblotting (lower panel A) as well as for 3 smokers with COPD (C) (lower panel B). Due to very low SP-D levels in BAL of COPD subjects the displayed intensity of SP-D bands is not comparable between A and B.

In addition to the quantitative changes of SP-D levels in the different compartments, its quaternary structure was analysed in BAL of healthy controls, smokers and patients with COPD (Figure 5A and 5B, bottom panels). Separation under native conditions (Figure 5A lower panel) did not show immunoreactive bands and therefore did not provide evidence for changes in the quaternary structure of SP-D from healthy, non smoking subjects (H). Note that intact SP-D is too large to migrate into the gel. In contrast, lower molecular weight SP-D bands could be detected in the SP-D of smoking subjects (S1), suggesting structural alterations of SP-D, possibly through the disruption of its quaternary structure towards smaller subunits.

Due to the much lower concentrations of SP-D levels in BAL of patients with COPD compared to healthy individuals (Figure 1A), samples were concentrated prior to analysis by native electrophoresis and immunoblotting. The quaternary structure of SP-D in smokers with COPD was also found disrupted (Figure 5B), however, due to the concentration process, the immunoblot shown in Figure 5B is not comparable to protein band intensities of S1 shown in Figure 5A.

**Figure 6:**
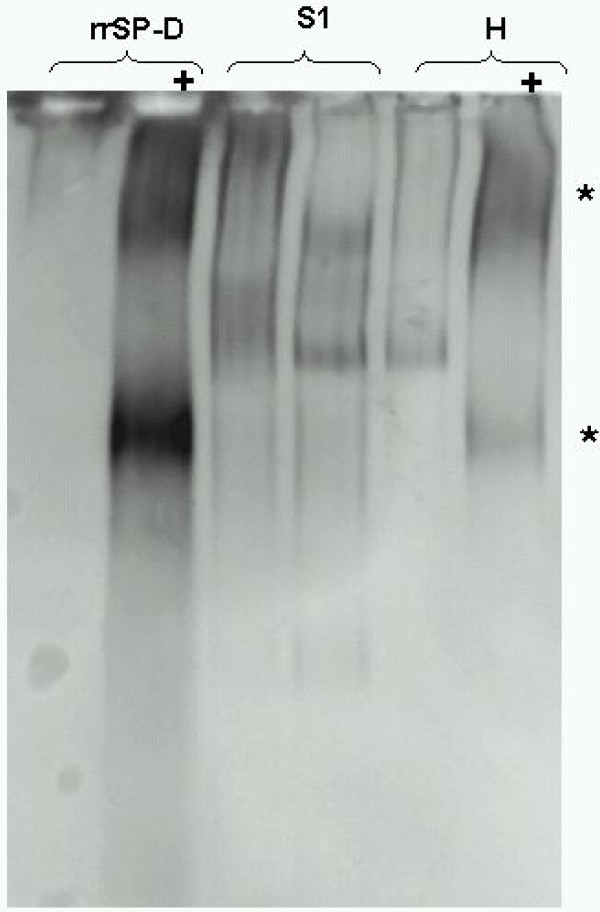
**Oxidative induced disruption of the quaternary structure of pulmonary SP-D**. Blue native electrophoresis of surfactant protein-D after incubation with an oxygen radical donor (+). Disruption of the multimeric structure (*) can be chemically induced by exposing rr-SP-D or BAL from healthy subjects to 74 mM of the oxidizing agent 2,2-Azo-bis-(2-amidinopropane)-dihydrochloride (+).

Since increased amounts of reactive oxygen species (ROS) are thought to be present in the lungs of smokers (either derived from cigarette smoke itself or subsequently induced by inflammatory cells) we tested, whether oxygen radicals can alter the structure of recombinant rat SP-D (rrSP-D) and SP-D in BAL of a healthy non-smoker (H) *in vitro*. As demonstrated in Figure 6, the incubation with an oxygen radical donor resulted in the disruption of the native structure of rrSP-D into smaller subunits. SP-D from BAL of healthy subjects also migrated into the gel after treatment with the oxygen radical donor, indicating that smaller subunits were present. However, the pattern of oxidized SP-D *in vitro *did not exactly match the SP-D band pattern found in smokers *in vivo *(S1, Figure 6) probably due to differences in processes that occur *in vitro *and *in vivo*.

## Discussion

Using samples simultaneously obtained from the two major reservoirs of SP-D, the current study presents new data demonstrating that pulmonary and serum SP-D levels appear to be stably expressed in both patients with COPD and controls, can be influenced by smoking, and reflect the degree of airway obstruction and disease state. The highest pulmonary and the lowest serum SP-D concentrations were detected in healthy subjects. Smoking reduced the level of SP-D in BAL and increased the concentration in serum, apparently independent of age and smoking history. In active smokers with COPD, changes in BAL/serum SP-D ratio were most pronounced and for the first time, within minutes after the start of moderate exercise, an increase in serum SP-D levels with a reproducible kinetic profile was observed in smokers and in patients with COPD. Finally, we found changes in the quaternary structure of SP-D in these two groups suggesting a previously unappreciated smoking related effect. Together the data support the hypothesis for the translocation of SP-D from airways to serum and underscore the importance of concentration gradients, barrier integrity, and potentially quaternary structure in influencing the quantitative expression levels in these compartments.

The majority of SP-D production occurs in the lung with spatial localization to type II pneumocytes and Clara cells lining the distal airways [25]. The fact that SP-D can also be detected in serum moved it into focus as a potential biomarker [12,26]. SP-D has been analysed in BAL and serum of different patient groups before, however so far, only indirect evidence suggests lower than normal values in BAL and higher than normal values in serum of COPD patients [12,16]. Our data show that the BAL/serum ratio is markedly changed in COPD patients. In our present study we demonstrate that drastic changes with respect to the BAL/serum ratio are especially evident in COPD patients. Under the conditions used in our study, the BAL/serum ratio was about 10-fold higher in healthy subjects as compared to COPD patients.

BAL fluid recovery from COPD patients is often potentially more variable than healthy volunteers, as indicated in Table 2. However, protein levels were not statistically different between groups making it rather unlikely that differences in BAL dilution are responsible for the observed differences in BAL SP-D concentrations. In addition, the measurement of SP-D in BAL showed a decent reproducibility and was in a similar range as described by Sims et al. [16]. The SP-D concentrations in serum showed an even better reproducibility and were comparable to those reported by Lomas et al. [12]. Furthermore, the stability of the BAL/serum ratios over a time period of 4 weeks indicates that this ratio reflects individual physiological conditions and disease states.

In line with previous studies we found a weak but significant correlation between SP-D levels in BAL and in serum with the degree of airway obstruction [26]. Interestingly our correlation confirm data from a recently published study, where the ratio of SP-D in BAL and serum was shown to correlate significantly with the degree of airway obstruction in a trial with smoking subjects [27]. These data are encouraging to use SP-D as a biomarker/surrogate marker for clinical read outs and would also justify larger validation trials.

The function of SP-D in serum if any as well as its source is still unclear. Serum SP-D levels have been shown to be steroid sensitive and to reflect an increased risk for exacerbations in patients with COPD [12]. The origin of serum SP-D is currently considered to be the lung and raised serum levels have been related to increased concentrations in the lung e.g. during allergic inflammation [14], or have been suggested to be due to an increased permeability of the lung and leakage from the pulmonary site [12]. The changes in the BAL/serum ratio observed in our study support the permeability hypothesis. However, the reproducible rapid increase during exercise in smokers and COPD patients in serum SP-D concentrations would then require a similar rapid change in alveolar/vascular permeability, which at least in healthy subjects [28] has not been detected before.

The similar protein concentration in BAL fluid between groups further suggest that the translocation of molecules between serum and the lung is rather complex. Our data does not solve this issue and it is important to keep in mind that SP-D is not only expressed in the lung and could therefore also be derived from other extrapulmonary sources [29].

Beside alterations of SP-D levels, we found remarkable changes in protein patterns after native separation and immunoblotting indicating a loss of its multimeric structure in smokers and smokers with COPD. The disruption of the multimeric structure of SP-D can have several deleterious consequences regarding its function in host defence and innate immunity [30], lowering the binding affinity to pathogen ligands [7] and might also reduce its anti-oxidant functions [6]. The loss of multimeric integrity towards smaller subunits might also play a role in the hypothesized increased translocation of pulmonary SP-D into the circulation because SP-D molecules with lower molecular weight might more easily translocate into the systemic circulation.

Although native gel electrophoresis does not allow a prediction of the precise molecular weight of a protein, we found SP-D fragments in BAL in a range of about 200-800 kDa, indicating that it was not completely disrupted to monomers (43 kDa) but rather de-multimerized. Such degradation products have not been observed or reported so far, and it is tempting to speculate that oxidative and nitrosative stress might be the causes to initiate disruption by amino acid modification. Whether the appearance of lower multimeric forms or the susceptibility to post-tranlational modification by SNO, ONOO or crosslinking of SP-D are affected by the DNA polymorphism Thr/Thr^11 ^described by Leth-Larsen remains speculative, since we did not analyse for polymorphisms [31]. Further, we found smaller multimeric forms in smokers and significantly increased SP-D serum levels, whereas Leth-Larsen reported an reduction of SP-D serum levels associated with Thr/Thr^11 ^genotypes.

Indeed, we found more nitrite in smoker compared to non-smoker BAL and first evidence for more S-nitrosylated SP-D (SNO-SP-D) in smokers (data not shown). The formation of S-nitrosothiol occurs through reactive nitrogen species and causes loss of multimerization and additional pro-inflammatory signalling activity on macrophages [9].

Radical oxygen species have also the potential to modify proteins, introducing carbonyl groups at certain amino acids [32]. This modification can alter the quaternary structure, and increase the susceptibility to further degradation by proteinases. ROS are elevated in smokers [33], derived either directly from inhaled cigarette smoke or are released in response to smoking from various cells like neutrophils and macrophages. We could show *in vitro *that the native structure of SP-D can be modified by an oxidant but we were not able to detect carbonyl groups in BAL from smokers due to methodical limitations. However, in cystic fibrosis these oxidative modifications of SP-D have been observed and they were associated with a loss of functional properties i.e. a reduced agglutination of *Pseudomonas aeruginosa *[18]. Increased bacterial and viral colonisation are common in patients with COPD [34], which might be linked to reduced pulmonary SP-D levels as well as to a potentially impaired functionality due to the observed disrupted structure [6]. To further elucidate the role of SP-D structural modifications in COPD, a quantitative evaluation between all groups with respect to the proportions of disrupted relative to the total SP-D level will be required, and there appears to be a need to clarify if ELISA measurements are affected by these SP-D modifications.

In conclusion, we showed that pulmonary and serum SP-D levels are stable markers that are related to smoking, airway obstruction, and disease state. In addition, we demonstrated that cigarette smoke is capable to disrupt SP-D's quaternary structure, which might play a role in an impaired immunological function and an increased translocation of SP-D from the lung into the circulation.

## Competing interests

The interpretation and presentation of these results does not influence the personal or financial relationship of any of the authors with other people or organisations.

Conception and design: CW, OH, VJE, ME, JMH

Acquisition of data: CW, ENAV, NK, SR, GL

Clinical study conduct: NK, JMH

Analysis and interpretation: CW, ENAV, OH, JMH

Drafting the manuscript for important intellectual content: CW, ENAV, JMH

Revision of the manuscript for important intellectual content: ENAV, OH, MFB, VJE, NK, SR, GL, ME

Final approval of the manuscript: all authors.

## References

[B1] Cosio MG Saetta M Agusti A Immunologic aspects of chronic obstructive pulmonary disease N Engl J Med 2009 360 2445 2454 10.1056/NEJMra0804752 19494220

[B2] Rabe KF Hurd S Anzueto A Barnes PJ Buist SA Calverley P Fukuchi Y Jenkins C Rodriguez-Roisin R van Weel C Zielinski J Global strategy for the diagnosis, management, and prevention of chronic obstructive pulmonary disease: GOLD executive summary Am J Respir Crit Care Med 2007 176 532 555 10.1164/rccm.200703-456SO 17507545

[B3] Wright JR Immunoregulatory functions of surfactant proteins Nat Rev Immunol 2005 5 58 68 10.1038/nri1528 15630429

[B4] Fisher JH Sheftelyevich V Ho YS Fligiel S McCormack FX Korfhagen TR Whitsett JA Ikegami M Pulmonary-specific expression of SP-D corrects pulmonary lipid accumulation in SP-D gene-targeted mice Am J Physiol Lung Cell Mol Physiol 2000 278 L365 L373 10666121 10.1152/ajplung.2000.278.2.L365

[B5] Kishore U Greenhough TJ Waters P Shrive AK Ghai R Kamran MF Bernal AL Reid KB Madan T Chakraborty T Surfactant proteins SP-A and SP-D: structure, function and receptors Mol Immunol 2006 43 1293 1315 10.1016/j.molimm.2005.08.004 16213021

[B6] Matalon S Shrestha K Kirk M Waldheuser S McDonald B Smith K Gao Z Belaaouaj A Crouch EC Modification of surfactant protein D by reactive oxygen-nitrogen intermediates is accompanied by loss of aggregating activity, in vitro and in vivo FASEB J 2009 23 1415 1430 10.1096/fj.08-120568 19126597 PMC2669423

[B7] Brown-Augsburger P Chang D Rust K Crouch EC Biosynthesis of surfactant protein D. Contributions of conserved NH2-terminal cysteine residues and collagen helix formation to assembly and secretion J Biol Chem 1996 271 18912 18919 10.1074/jbc.271.31.18912 8756121

[B8] Gardai SJ Xiao YQ Dickinson M Nick JA Voelker DR Greene KE Henson PM By binding SIRPalpha or calreticulin/CD91, lung collectins act as dual function surveillance molecules to suppress or enhance inflammation Cell 2003 115 13 23 10.1016/S0092-8674(03)00758-X 14531999

[B9] Guo CJ Atochina-Vasserman EN Abramova E Foley JP Zaman A Crouch E Beers MF Savani RC Gow AJ S-nitrosylation of surfactant protein-D controls inflammatory function PLoS Biol 2008 6 e266 10.1371/journal.pbio.0060266 19007302 PMC2581630

[B10] Honda Y Kuroki Y Matsuura E Nagae H Takahashi H Akino T Abe S Pulmonary surfactant protein D in sera and bronchoalveolar lavage fluids Am J Respir Crit Care Med 1995 152 1860 1866 8520747 10.1164/ajrccm.152.6.8520747

[B11] Ohnishi H Yokoyama A Kondo K Hamada H Abe M Nishimura K Hiwada K Kohno N Comparative study of KL-6, surfactant protein-A, surfactant protein-D, and monocyte chemoattractant protein-1 as serum markers for interstitial lung diseases Am J Respir Crit Care Med 2002 165 378 381 11818324 10.1164/ajrccm.165.3.2107134

[B12] Lomas DA Silverman EK Edwards LD Locantore NW Miller BE Horstman DH Tal-Singer R Serum surfactant protein D is steroid sensitive and associated with exacerbations of COPD Eur Respir J 2009 34 95 102 10.1183/09031936.00156508 19164344

[B13] Hermans C Bernard A Lung epithelium-specific proteins: characteristics and potential applications as markers Am J Respir Crit Care Med 1999 159 646 678 9927386 10.1164/ajrccm.159.2.9806064

[B14] Erpenbeck VJ Schmidt R Gunther A Krug N Hohlfeld JM Surfactant protein levels in bronchoalveolar lavage after segmental allergen challenge in patients with asthma Allergy 2006 61 598 604 10.1111/j.1398-9995.2006.01062.x 16629790

[B15] Koopmans JG van der Zee JS Krop EJ Lopuhaa CE Jansen HM Batenburg JJ Serum surfactant protein D is elevated in allergic patients Clin Exp Allergy 2004 34 1827 1833 10.1111/j.1365-2222.2004.02083.x 15663555

[B16] Sims MW Tal-Singer RM Kierstein S Musani AI Beers MF Panettieri RA Haczku A Chronic obstructive pulmonary disease and inhaled steroids alter surfactant protein D (SP-D) levels: a cross-sectional study Respir Res 2008 9 13 10.1186/1465-9921-9-13 18226251 PMC2249580

[B17] Hirche TO Crouch EC Espinola M Brokelman TJ Mecham RP DeSilva N Cooley J Remold-O'Donnell E Belaaouaj A Neutrophil serine proteinases inactivate surfactant protein D by cleaving within a conserved subregion of the carbohydrate recognition domain J Biol Chem 2004 279 27688 27698 10.1074/jbc.M402936200 15078883

[B18] Starosta V Griese M Oxidative damage to surfactant protein D in pulmonary diseases Free Radic Res 2006 40 419 425 10.1080/10715760600571248 16517507

[B19] Hoegh SV Sorensen GL Tornoe I Lottenburger T Ytting H Nielsen HJ Junker P Holmskov U Long-term stability and circadian variation in circulating levels of surfactant protein D Immunobiology 2010 215 314 320 10.1016/j.imbio.2009.05.001 19540617

[B20] Thum T Erpenbeck VJ Moeller J Hohlfeld JM Krug N Borlak J Expression of xenobiotic metabolizing enzymes in different lung compartments of smokers and nonsmokers Environ Health Perspect 2006 114 1655 1661 17107849 10.1289/ehp.8861 PMC1665420

[B21] Beirne P Pantelidis P Charles P Wells AU Abraham DJ Denton CP Welsh KI Shah PL du Bois RM Kelleher P Multiplex immune serum biomarker profiling in sarcoidosis and systemic sclerosis Eur Respir J 2009 19541722 10.1183/09031936.00028209

[B22] Atochina EN Beck JM Preston AM Haczku A Tomer Y Scanlon ST Fusaro T Casey J Hawgood S Gow AJ Beers MF Enhanced lung injury and delayed clearance of Pneumocystis carinii in surfactant protein A-deficient mice: attenuation of cytokine responses and reactive oxygen-nitrogen species Infect Immun 2004 72 6002 6011 10.1128/IAI.72.10.6002-6011.2004 15385504 PMC517574

[B23] Schagger H von Jagow G Blue native electrophoresis for isolation of membrane protein complexes in enzymatically active form Anal Biochem 1991 199 223 231 10.1016/0003-2697(91)90094-A 1812789

[B24] Crouch E Persson A Chang D Heuser J Molecular structure of pulmonary surfactant protein D (SP-D) J Biol Chem 1994 269 17311 17319 8006040

[B25] Mori K Kurihara N Hayashida S Tanaka M Ikeda K The intrauterine expression of surfactant protein D in the terminal airways of human fetuses compared with surfactant protein A Eur J Pediatr 2002 161 431 434 10.1007/s00431-002-0917-9 12172826

[B26] Sin DD Leung R Gan WQ Man SP Circulating surfactant protein D as a potential lung-specific biomarker of health outcomes in COPD: a pilot study BMC Pulm Med 2007 7 13 10.1186/1471-2466-7-13 17922919 PMC2096624

[B27] Tkacova R McWilliams A Lam S Sin DD Integrating lung and plasma expression of pneumo-proteins in developing biomarkers in COPD: a case study of surfactant protein D Med Sci Monit 2010 16 CR540 CR544 20980958

[B28] Hoegh SV Sorensen GL Tornoe I Lottenburger T Ytting H Nielsen HJ Junker P Holmskov U Long-term stability and circadian variation in circulating levels of surfactant protein D Immunobiology 2010 215 314 320 10.1016/j.imbio.2009.05.001 19540617

[B29] Madsen J Kliem A Tornoe I Skjodt K Koch C Holmskov U Localization of lung surfactant protein D on mucosal surfaces in human tissues J Immunol 2000 164 5866 5870 10820266 10.4049/jimmunol.164.11.5866

[B30] Atochina-Vasserman EN Beers MF Gow AJ Review: Chemical and structural modifications of pulmonary collectins and their functional consequences Innate Immun 2010 16 175 182 10.1177/1753425910368871 20423921 PMC4361894

[B31] Leth-Larsen R Garred P Jensenius H Meschi J Hartshorn K Madsen J Tornoe I Madsen HO Sorensen G Crouch E Holmskov U A common polymorphism in the SFTPD gene influences assembly, function, and concentration of surfactant protein D J Immunol 2005 174 1532 1538 15661913 10.4049/jimmunol.174.3.1532

[B32] Davies KJ Delsignore ME Lin SW Protein damage and degradation by oxygen radicals. II. Modification of amino acids J Biol Chem 1987 262 9902 9907 3036876

[B33] Walser T Cui X Yanagawa J Lee JM Heinrich E Lee G Sharma S Dubinett SM Smoking and lung cancer: the role of inflammation Proc Am Thorac Soc 2008 5 811 815 10.1513/pats.200809-100TH 19017734 PMC4080902

[B34] Rosell A Monso E Soler N Torres F Angrill J Riise G Zalacain R Morera J Torres A Microbiologic determinants of exacerbation in chronic obstructive pulmonary disease Arch Intern Med 2005 165 891 897 10.1001/archinte.165.8.891 15851640

